# Evaluation of *Candida albicans* biofilm formation on conventional and computer-aided-design/computer-aided manufacturing (CAD/CAM) denture base materials

**DOI:** 10.18502/cmm.8.3.11208

**Published:** 2022-09

**Authors:** Milad Larijani, Zahra Zareshahrabadi, AbdolHamid Alhavaz, Romina Hajipour, Alireza Ranjbaran, Rashin Giti, Vahid Soltankarimi, Kamiar Zomorodian

**Affiliations:** 1 Dental Materials Research Center, Health Research Institute, Babol University of Medical Sciences, Babol, Iran; 2 Basic Sciences in Infectious Diseases Research Center, Shiraz University of Medical Sciences, Shiraz, Iran; 3 Dental Materials Research Center, Health Research Institute, Babol University of Medical Sciences, Babol, Iran; 4 Resident, Department of Endodontic, Islamic Azad University, Dental School, Khorasegan Branch, Isfahan, Iran; 5 Oral and Dental Disease Research Center, Department of Oral and Maxillofacial Medicine, School of Dentistry, Shiraz University of Medical Sciences, Shiraz, Iran; 6 Department of Prosthodontics, Biomaterials Research Center, School of Dentistry, Shiraz University of Medical Sciences, Shiraz, Iran; 7 Department of Parasitology and Mycology, School of Medicine, Shiraz University of Medical Sciences, Shiraz, Iran; † These authors contributed equally to this work

**Keywords:** Adhesion, Biofilm, *Candida albicans*, Denture

## Abstract

**Background and Purpose:**

The human mouth mucosal surface is colonized by indigenous microflora, which normally maintains an ecological balance among different species. However, certain environmental or biological factors may disrupt this balance, leading to microbial diseases. *Candida albicans* biofilms are formed on indwelling medical devices and have an association with both oral and invasive candidiasis. This study aimed to compare the amount of adherent *C. albicans* and the biofilm formed on different denture base materials. The adhesion of *C. albicans* to denture base materials is widely recognized as the main reason for the development of denture stomatitis.

**Materials and Methods:**

In total, 56 polymethyl methacrylate (PMMA) acrylic resin disc-shaped samples were divided into four groups as follows: 1) chemically polymerized PMMA, 2) heat-polymerized PMMA, 3) computer-aided design and computer-aided manufacturing (CAD/CAM) PMMA in high polish, and 4) CAD/CAM resins in glazed form. The adherent cells and formation of *C. albicans* strains (562, 1905, 1912, and 1949) biofilm were measured by the 2,3-bis-(2-methoxy-4-nitro-5-sulfophenyl)-2H-tetrazolium-5-carboxanilide (XTT) method and use of a microplate reader. Moreover, morphological alterations of *C. albicans* cells were investigated using scanning electron microscopy (SEM)

**Results:**

The biofilm formation was significantly lower on CAD/CAM acrylic resins, compared to conventional denture base materials. The obtained results were confirmed by the SEM images of C. albicans biofilms. CAD/CAM PMMA-based polymers may be preferable to inhibit C. albicans biofilm formation and reduce Candida-associated denture stomatitis in long-term use.

**Conclusion:**

Based on the findings, the CAD/CAM technique can be used as an efficient technique for denture fabrication as it inhibits microbial accumulation, and consequently, microbial biofilm.

## Introduction

Denture stomatitis is a multifactorial disease and can be caused by many predisposing factors. Some of these factors are related to underlying systemic and immune diseases, as well as a deficiency in salivary flow rate, while others are specific to denture wearers, such as poor denture hygiene. Moreover, it can be caused by roughness or the presence of pores in the acrylic surface, which can result in denture-induced trauma. Denture hygiene and oral hygiene are very important factors in controlling the colonization of microorganisms on the denture surface [ 1 - 3 ]. According to previous epidemiological studies, the prevalence rate of denture stomatitis is 15%-70% among denture wearers [ 4 ].

Among the mentioned factors, microbial colonization in the oral cavity and and on the surface of dentures is well-known as a predisposing factor in denture wearers, which can cause denture stomatitis. *Candida* is a major part of the normal commensal flora in the oral cavity. A change in the oral microflora, followed by an increase in *Candida* colonization, which is the most common manifestation of oral candidiasis, can cause *Candida*-associated denture stomatitis in denture wearers [ 5 , 6 ].

*Candida albicans* is the most prevalent species of *Candida*, followed by *C. glabrata*, *C. krusei*, *C. kefyr*, *C. parapsilosis*, and *C. tropicalis* [ 7 ]. *C. albicans* can adhere and grow on the oral mucosa and surfaces of the acrylic resins of dentures, causing biofilm formation and oral infection. Biofilm formation, as a protective reservoir, plays an important role in the inhibition of host clearance mechanisms and can cause drug resistance in the oral cavity [ 8 , 9 ].

Based on previous studies, there is an association between the characteristics of the denture acrylic surface and the amount of *Candida* colonization, followed by biofilm formation [ 10 , 11 ]. Other studies have also confirmed that surface roughness, porosity, and permeability can affect *Candida* colonization and biofilm formation on the removable denture acrylic surface [ 12 , 13 ].

Previous studies reviewed the predisposing factors involved in microbial attachment and colonization on the denture, such as the surface characteristics of the acrylic resin, microorganism strain, and subsequently, the interaction of these two factors in attachment and colonization on the denture surfaces [ 14 , 15 ].

Polymethyl methacrylate (PMMA) is the most popular and available material for making prosthetic teeth and dentures. According to previous studies, conventional acrylic dentures have disadvantages, such as low mechanical strength, fractures after a few years of usage, polymerization shrinkage, allergic reactions mainly due to monomer saturation, degradation of mechanical properties, and lack of resistance to corrosion in the environment, especially in human saliva. It is noteworthy that the type of curing can influence the number of pores in the denture surface and susceptibility to microbial colonization and biofilm formation [ 15 , 16 ]. Computer-aided design and computer-aided manufacturing (CAD/CAM) techniques have been available for a while, and their usage continues to increase in the field of prosthodontics.

In the CAD/CAM technique, the denture bases are milled from industrially polymerized resin pucks due to the high pressure that is applied during polymerization. Therefore, they are less likely to be porous and have a higher degree of polymerization. In addition, the completely automated process produces smoother surfaces in dentures in comparison with conventional manual fabrication [ 17 ]. In this regard, the present study aimed to compare the *C. albicans* biofilm formation on the surfaces of conventionally and CAD/CAM fabricated denture base materials.

## Materials and Methods

This study was performed on 56 acrylic resin discs with different processing techniques. The samples were divided into four groups of 14 discs as follows: 1) chemically polymerized PMMA, 2) heat-polymerized PMMA, 3) CAD/CAM resins with a high polish, and 4) CAD/CAM resins with a surface glaze. 

### Construction of conventional acrylic resin

To make chemically cured PMMA (Ivoclar Vivadent, Schaan, Liechtenstein) samples, 10×1 mm of dental waxes were implanted in cuvettes with dental plaster. According to the manufacturer's instructions, after setting them, the waxes were removed. Subsequently, the acrylic powder and liquid were mixed and packed into the prepared generators and kept at room temperature until the polymerization process was completed.

Likewise, heat-polymerized acrylic (Ivoclar Vivadent, Schaan, Liechtenstein) discs were created and placed inside stainless-steel molds. Afterward, the flashing process was carried out. The boiling process was performed for nine hours at a constant temperature of 73.5°C. In the next stage, the prepared samples were examined, and the samples containing bubbles, incomplete polymerization, or any irregularity were excluded from the study. 

### Construction of acrylic resin using computer-aided design and computer-aided manufacturing

To prepare denture samples by CAD/CAM (AD; Global Dental Science Europe BV, Tilburg, the Netherlands), the specimens were designed in CAD software (Remote DENTAL 2.0; imes-icore GmbH, Hamburg, Germany). Afterward, the data were transferred to a CAD/CAM machine (iMES-iCORECORiTEC 340i; Hamburg, Germany) to prepare acrylic resin samples of the same size (10×1 mm). In the high-polish CAD/CAM group, polishing was performed with a polishing compact unit (Derotor, London, England) consisting of a polishing lathe, a 45-mm polishing brush, and a pleated buff nettle cloth (Renfert GmbH, Industrie-gebiet, Hilzingen, Germany) with pumice (Pumice CL 60, Coarse Grade, Whip Mix Corporation, Louisville, KY, USA). For the surface glazed CAD/CAM group, optiglaze (OPTIGLAZE, GC, America) was applied on the surface of specimens after polishing according to the manufacturer's instructions. 

All the samples were stored in a flask containing 90% ethanol for one minute, washed in running distilled water, and immersed in sterile water for 24 h at 24°C [ 8 ].

### Preparation of Candida albicans suspension

The four standard strains of *C. albicans* (CBS 562, CBS 1905, CBS 1912, and CBS 1949) were purchased from the Centraal Bureau voor Schimmelcultures, ([CBS], Utrecht, the Netherlands) and cultured on sabouraud dextrose agar (Merck, Germany). After 24 h, a loopful of the fresh yeast colonies was transferred to 20 mL sabouraud dextrose broth (Merck, Germany) and incubated at 30°C on the shaker at 100 rpm (24h). Subsequently, the yeast cells were harvested by centrifugation at 3000 g for 10 min and washed twice in sterile phosphate-buffered saline (PBS) (0.8% [w/v], NaCl [Merck, Germany]; 0.02% [w/v], KH_2_PO_4_ [Merck, Germany]; 0.31% [w/v], Na_2_HPO_4_+12H_2_O (Merck, Germany); 0.02% [w/v], KCl [Panreac, Spain]; pH: 7.4). Afterward, the washed *C. albicans* cells were re-suspended in RPMI-1640 (Sigma, USA) media with 50 mmol^−1^ glucose. The cell densities were adjusted to 1.0×10^8^ cells/mL and 0.15 optical densities at a wavelength of 530 nm. 

### Biofilm formation assays

#### Biofilm preparation and growth

In this study, biofilm formation inhibition was determined using a technique previously described by Ramage *et al*. with some modification [ 18 ]. In total, 14 acrylic resin samples from each group were used for the biofilm formation assay. Prior to the tests, the samples were autoclaved at 121°C and exposed to ultraviolet light in dry conditions at room temperature for 20 min to kill any microorganisms that may have contaminated the specimens during fabrication or storage. 

The resulting *C. albicans* suspensions were diluted 1/1000 by RPMI-1640 medium; afterward, 500 μL of the standardized *C. albicans* suspensions were added to each well of a 24-well tissue culture plate (Corning, St. Louis, MO, USA) containing the acrylic resin samples. Subsequently, the plates were incubated for 48 h at 37°C. In addition, 500 μL of the RPMI-1640 medium was used as the negative control (blank); however, the RPMI-1640 with the yeasts and without the *acrylic resin samples were* considered the positive controls. It should be mentioned that all experiments were performed in triplicate [ 19 ].

#### Quantitative measurement of Candida albicans biofilm

The metabolic activity of *C. albicans* biofilms was calculated using a colorimetric assay. Biofilm formation was performed using a 2,3-bis (2-methoxy-4-nitro-5-sulfo-phenyl)-2H-tetrazolium-5-carbox-anilide (XTT) (Sigma, St Louis, MO, USA) reduction assay as an indicator of cell survival and proliferation, which was prepared in Ringers lactate solution (0.5 mg/mL). 

The solution was filter-sterilized (0.22μm-pore-size) and stored at -70°C. Prior to each assay, the XTT stock solution was mixed with menadione sodium bisulfite (10 mM, Sigma Chemical Co., St. Louis, USA). After 48 h of incubation, the discs were transferred to a new tissue culture plate and washed twice with sterile PBS. Subsequently, a 500 μL aliquot of XTT/menadione was added to each well of the 24-well plates. The plates were incubated at 37°C in a dark room for 3 h. Finally, the colorimetric changes were measured at 570 nm using a microplate reader (BMG Labtech, Berlin, Germany). The biofilm formation was calculated as follows: biofilm formation percent=(absorbance of test well/absorbance of growth control well)×100 [ 19 ]. 

#### Qualitative observation of the Candida albicans biofilm

Scanning electron microscopy (SEM) was used to observe the biofilm that had formed on the surfaces. To examine the ultrastructural nature of grown biofilms and the morphological features of *C. albicans*, acrylic resin denture base samples were fixed in 2.5% glutaraldehyde in 0.1 M phosphate buffer (pH 7.2) at 4°C for 1 h.

After being washed in buffer, the samples were postfixed in 1% osmium tetroxide in the same buffer for 30 min. The samples were dehydrated in the graded concentrations of ethanol and critical point-dried in CO2 (Polaron Critical Point Dryer). They were then coated with colloidal gold (Balzers SCD 050 Sputter Coater, Baltic, Liechtenstein) and viewed under a Leo 435 VP SEM (Oxford Instruments, Oxford, UK) at 15 kV [ 9 ].

### Statistical analysis of data

Descriptive statistics were used to describe the *C. albicans* biofilm formation values for the conventional and CAD/CAM denture base materials. The quantitative data were presented as mean and standard deviation. A one-way ANOVA test was used to compare the biofilm formation of the four *C. albicans* strains between these types of complete denture bases. It should be noted that a P-value of less than 0.05 was considered statistically significant. 

## Results

Table 1 summarizes the biofilm formation by XTT reduction assay in the four mentioned groups. The mean optical density of *C. albicans* biofilm formation was reported to be 0.255, 0.129, 0.124, and 0.086 in the studied groups, including 1) chemically-polymerized PMMA, 2) heat-polymerized PMMA, 3) CAD/CAM resins with surface glaze, and 4) CAD/CAM resins with a high polish, respectively.

**Table 1:** Optical density and percent of *Candida albicans* biofilm formation on different types of acrylic resin by XTT reduction assay.

*C. albicans* Strains (CBS)	Conventional acrylic resins				CAD/CAM acrylic resins			
	Chemically polymerized		Heat-polymerized		Glazed		High polished	
	OD±SD	Biofilm formation (%)±SD	OD±SD	Biofilm formation (%)±SD	OD±SD	Biofilm formation (%)±SD	OD±SD	Biofilm formation (%)±SD
562	0.25±0.032	62.5±15.6	0.15±0.018	37.5±11.2	0.12±0.018	30±14.2	0.08±0.014*	20.2±8.2
1905	0.28±0.031	70±16.8	0.13±0.015	28.5±12.5	0.13±0.014	32.5±10.2	0.09±0.016*	22.5±10.5
1912	0.26±0.029	65.3±21.1	0.13±0.015	28.5±13.1	0.12±0.016	30±9.5	0.12±0.015*	30.6±11.2
1949	0.31±0.03	77.2±19.2	0.12±0.014	30±12.4	0.14±0.015	35±12.2	0.07±0.017*	17.5±8.5

Note: OD value mean with superscript letters (*) differ significantly (P<0.001). OD: Optical density, SD: Standard Deviation, CBS: CBS: Centraal Bureau voor Schimmelcultures, CAD/CAM: computer-aided design and computer-aided manufacturing.

According to Table 1, all acrylic resin discs showed antibiofilm activity and were able to reduce biofilm formation in comparison with growth control. Statistical analysis of the results using a one-way ANOVA test showed that the mean and standard deviation of *C. albicans* biofilm formation decreased significantly in CAD/CAM polished acrylic denture samples (*P<0.001*) (Table 1).

**Figure 1:**
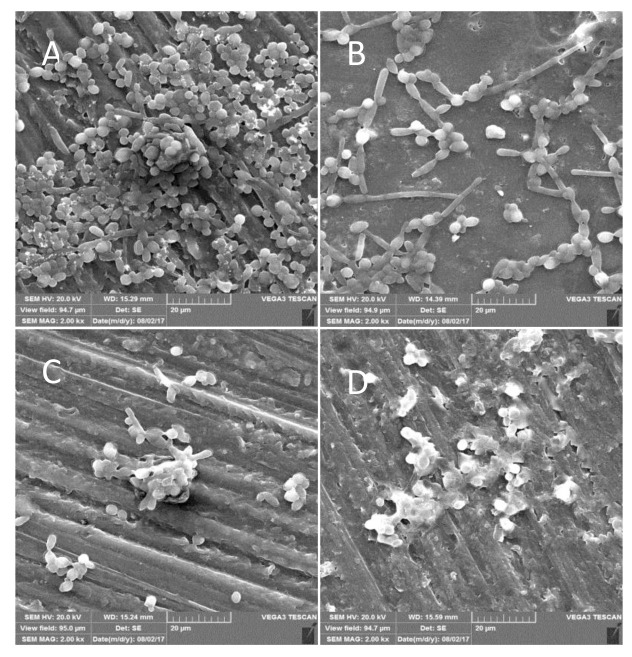
Scanning electron microscope images (×2000 magniﬁcation) of *C. albicans* biofilms on the denture resin specimen's base. A: chemically-polymerized poly methyl methacrylate (PMMA); B. heat-polymerized PMMA; C: computer-aided design and computer-aided manufacturing (CAD/CAM) resins with high polish; D: CAD/CAM resins with surface glaze

There was no significant difference between the biofilm formation of the heat-polymerized acrylic and CAD/CAM glazed denture samples. It is noteworthy that, in this study , significantly higher absorbance values were observed for the chemically polymerized PMMA acrylic samples in comparison with other types of acrylic resin. As shown in Table 1, the biofilm formation was inhibited in polished acrylic denture samples by up to 77%. Indeed, polished acrylic denture samples exhibited a significant activity in the inhibition of biofilm formation, as reflected in the lower absorbance reading and biofilm formation percent, compared to conventional acrylic resins. SEM evaluated the morphological alteration of *C. albicans* after biofilm formation in the mentioned group. As shown in Figure 1, SEM images revealed that the CAD/CAM acrylic resin was able to reduce *C. albicans* biofilm formation, compared to conventional acrylic resins. Fungal cells were found to a large extent on the surfaces of the conventional acrylic resins in SEM analysis. The morphology of the *C. albicans* biofilm showed that the CAD/CAM acrylic resins had an inhibitory effect on biofilm formation. The biofilm architecture was seriously damaged in the CAD/CAM high-polished resin forms, and few *C. albicans* cells were observed on these acrylic resins.

## Discussion

Despite the extreme increase in the use of dental implants, complete dentures are still considered a valuable treatment option for some edentulous patients who cannot afford or refuse implant placement [ 20 ].

According to previous studies, *Candida*-associated denture stomatitis is a common disease in denture wearers, and various *Candida* strains have been isolated from them [ 21 , 22 ]. According to various laboratory studies, *C. albicans* is the most frequently isolated strain that can adhere and form biofilms on the oral mucosa and subsequently cause denture stomatitis [ 22 , 23 ]. Over the decades, many efforts have been made to improve the material, the curing techniques, and the processes used for fabricating denture bases [ 10 , 15 ].

In the present study, *C. albicans* exhibited differing adherence and biofilm formation abilities regarding the CAD/CAM and conventionally fabricated complete denture bases. There was a significant difference between biofilm formation on the conventional and CAD/CAM denture samples. Statistical analysis of the results revealed that CAD/CAM polished acrylic denture samples had the least amount of biofilm formation. These findings suggested that yeast cells were less likely to adhere to CAD/CAM denture bases than to conventional denture bases. The fact that surface roughness and porosity are important factors in microbial adhesion and colonization may explain the lower adherence [ 24 ]. CAD/CAM groups exhibited more favorable surface properties, including a lower error rate, greater precision, better consistency, and greater adaptability, compared to conventional groups, according to previous research [ 16 , 24 , 25 ]. In this study, the polishing process significantly decreased the adherence of *C. albicans* to the surface of the dentures. Based on the findings of this study, the acrylic resin manufactured by CAD/CAM technology, along with the polishing process had the least amount of *C. albicans* adherence, which is in line with the results of previous studies [ 26 , 27 ]. 

In previous studies, the advantages of CAD/CAM technology with the polishing methods over conventionally fabricated complete dentures have been verified [ 14 ] which were also confirmed in the present research. In this study, the high biofilm formation rate on the chemically polymerized acrylic resin surfaces may be due to the porosity of the conventional acrylic resin denture bases as an unfavorable result.

It can be caused by air trapped during mixing, insufficient mixing of polymer and monomer, the existence of residual monomer, and inadequate compression on the flask, which may affect the adherence of microorganisms on the surface of the denture and increase microorganism colonization [ 26 , 27 ]. Besides, due to the use of pre-polymerized acrylic resin under special conditions, such as high pressure and temperature, the CAD/CAM base denture releases less monomer than the conventional form.

As a result, the CAD/CAM fabricated denture bases have a less porous and rough surface compared to conventional acrylic resin [ 15 ]. Therefore, the adhesion and biofilm formation of *C. albicans* and other oral microorganisms to the dentures fabricated using the CAD/CAM technique are less than those fabricated by the conventional denture processing methods [ 13 ]. One reason for this lower adhesion could be the hydrophobic surfaces of conventional acrylic resin bases, because monomers exposed on the surface of the denture may attach to microorganisms and form microbial biofilms. [ 28 , 29 ]. 

According to the results of the present and previous studies [ 13 , 30 ], the tension of the used materials increases precision in the dentures fabricated using the CAD/CAM techniques and creates smoother surfaces that decrease *C. albicans* adhesion and biofilm formation on the surfaces of the dentures. According to some previous studies, conventional complete denture bases have higher microbial colonization, and consequently, significant adhesion and biofilm formation of *C. albicans* in comparison with CAD/CAM complete dentures [ 16 , 31 ]. This reveals the superiority of CAD/CAM technology in the construction of denture bases and is in line with the results of the present study.

Agraval *et al*. conducted an *in vitro* study in 2015 about the *C. albicans* biofilm formation on different types of denture base materials. The findings of the aforementioned study revealed that cobalt-chrome alloy is the least adhesive, while heat-cured acrylic resin and chemically cured acrylic resin have shown the most adhesiveness, in that order [ 30 ]. Their results proved that the conventional method of fabricating acrylic resin has disadvantages that are addressed by modern technologies in dentistry, such as CAD/CAM.

Kattadiyil MT *et al*. performed a review study to assess the advantages of the CAD/CAM method in denture manufacturing. Similar to this study, their results also indicated that the denture material used in CAD/CAM technology has better properties, compared to the common chemically and heat-cured materials [ 32 ].

In the present research, the XTT assay was used to measure biofilm formation on acrylic resins since it has a high quantitative level and is more accurate in comparison with other methods used in previous studies [ 16 , 32 ]. SEM could be used as a semi-quantitative technique for fungal micromorphology evaluation since it allows the observation of interactions between the *Candida* and surface [ 8 ]. Hence, in this study, the findings of the SEM confirmed the results obtained from the XTT assay. In electronic microscope images, *C. albicans* biofilms appear mostly in the fissures and pores of the chemically polymerized denture base. 

As shown in the A and B images of Figure 1, the formation of blastoconidia, pseudohyphae, and hyphae was seen particularly in chemically polymerized acrylic resins fabricated using conventional methods. These structures are related to the biofilm formation and pathogenicity of *C. albicans* since they become a barrier against phagocytosis. These results were also in line with those of a study conducted by Gondim *et al*. [ 33 ].

## Conclusion

Based on the results, complete dentures fabricated using CAD/CAM procedure showed hopeful potential for reducing the *C. albicans* adherence and biofilm formation on the denture base surfaces. Finally, according to the findings, CAD/CAM technology can be suggested as the first choice for denture fabrication since it helps prevent microbial infection in dental settings.

## Acknowledgments

The authors wish to thank Mr. H. Argasi at the Research Consultation Center (RCC) of Shiraz University of Medical Sciences for his invaluable assistance in editing this manuscript.

## Authors’ contribution

K. Z. and V. S. K. designed and managed the present research. Z. Z. performed the tests, analyzed the data, and wrote the first draft of the manuscript. V. L., A. A., and A. R. edited the final manuscript. R. G. and R. H. were project partners. All authors approved the final version of the manuscript. 

## Conflicts of interest

The authors declare that they have no conflicts of interest.

## Financial disclosure

This study was part of the Milad Larijani's thesis.
